# Effective implementation of research into practice: an overview of systematic reviews of the health literature

**DOI:** 10.1186/1756-0500-4-212

**Published:** 2011-06-22

**Authors:** Annette Boaz, Juan Baeza, Alec Fraser

**Affiliations:** 1 Department of Primary Care and Public Health Sciences, King's College London, 7th Floor, Capital House, 42 Weston Street, London SE1 3QD, UK; 2 Department of Management, School of Social Science and Public Policy, King's College London, Franklin-Wilkins Building, 150 Stamford Street, London SE1 9NH, UK; 3 Department of Management, School of Social Science and Public Policy, King's College London, Franklin-Wilkins Building, 150 Stamford Street, London SE1 9NH, UK

## Abstract

**Background:**

The gap between research findings and clinical practice is well documented and a range of interventions has been developed to increase the implementation of research into clinical practice.

**Findings:**

A review of systematic reviews of the effectiveness of interventions designed to increase the use of research in clinical practice. A search for relevant systematic reviews was conducted of Medline and the Cochrane Database of Reviews 1998-2009. 13 systematic reviews containing 313 primary studies were included. Four strategy types are identified: audit and feedback; computerised decision support; opinion leaders; and multifaceted interventions. Nine of the reviews reported on multifaceted interventions. This review highlights the small effects of single interventions such as audit and feedback, computerised decision support and opinion leaders. Systematic reviews of multifaceted interventions claim an improvement in effectiveness over single interventions, with effect sizes ranging from small to moderate. This review found that a number of published systematic reviews fail to state whether the recommended practice change is based on the best available research evidence.

**Conclusions:**

This overview of systematic reviews updates the body of knowledge relating to the effectiveness of key mechanisms for improving clinical practice and service development. Multifaceted interventions are more likely to improve practice than single interventions such as audit and feedback. This review identified a small literature focusing explicitly on getting research evidence into clinical practice. It emphasizes the importance of ensuring that primary studies and systematic reviews are precise about the extent to which the reported interventions focus on changing practice based on research evidence (as opposed to other information codified in guidelines and education materials).

## Background

Despite significant investment in health research, challenges remain in translating this research into policies and practices that improve patient care. The gap between research findings and clinical practice is well documented [1,2] and a range of interventions has been developed to increase the implementation of research into health policy and practice. In particular, clinical guidelines, audit and feedback, continuing professional education and financial incentives are widely used and have been extensively evaluated [3].

Systematic reviews of existing research provide a rigorous method for assessing the relative effectiveness of different interventions that seek to implement research evidence into healthcare practice. A review by Grimshaw et al. [4] identified a range of strategies for changing provider behaviour ranging from educational interventions, audit and feedback, computerised decision support to financial incentives and combined interventions. The authors concluded that all the interventions had the potential to promote the uptake of evidence in practice, although no one intervention seemed to be more effective than the others in all settings.

This overview of systematic reviews of the health literature on the effectiveness of currently used implementation methods in translating research findings in to practice provides a focused update of Grimshaw et al.'s 2001 review. We detect a growing assumption that interventions designed to improve clinical practice and service development are always based on the best quality evidence, something that the pioneers of evidence-based medicine went to great lengths to point out was not (and was never likely to be) the case. We investigate whether any methods were effective in implementing research evidence. We excluded systematic reviews focusing on achieving change that were not sufficiently explicit about their evidence base. We want to know the effectiveness of implementation methods in translating evidence-based findings into practice as opposed to other non-evidenced-based changes.

## Methods

### Searches

**Figure 1:**
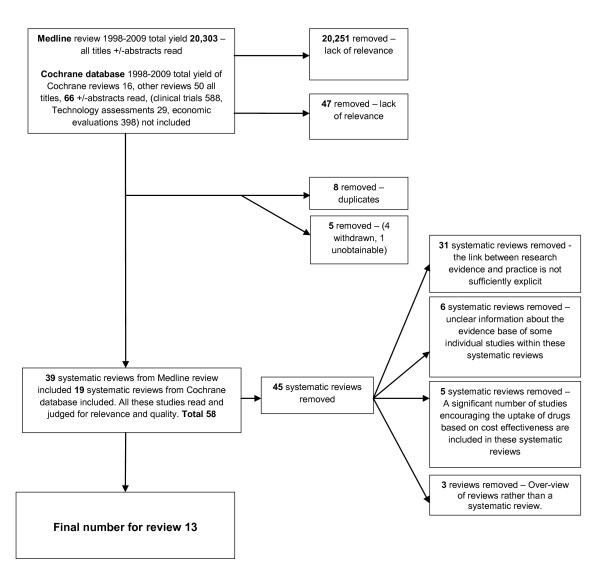
**Data extraction information**.

We searched Medline and the Cochrane Database of Reviews 1998-2009 using the search strategy employed by Grimshaw et al. [4]. Searches from 1966-July 1998 were completed by Grimshaw et al for their earlier review [4]. Full details of the data extraction process are given in Figure 1.

### Inclusion criteria

Systematic reviews are conducted to a set of consistent, transparent quality standards. As such, only systematic reviews were included in the review. In line with Franke et al. [5] reviews were considered to be systematic reviews if they met at least two of the following criteria: search terms were included; search included Pubmed/Medline; the methodological quality of the studies was assessed as part of the review. We included reviews that focused on the implementation of research evidence into practice. Study populations included healthcare providers and patients. Numerous interventions were assessed including clinical guidelines, audit and feedback, continuing professional education, financial incentives, use of opinion leaders and multifaceted interventions. Some systematic reviews included comparisons of different interventions, other reviews compared one type of intervention against a control group. Outcomes related to improvements in process or patient well-being. Numerous individual study types (RCT, CCT, BA, ITS) were included within the systematic reviews (see Additional file 1).

### Exclusion criteria

We excluded systematic reviews that did not look explicitly at interventions designed to get research evidence into practice [6]. However, this is far from straightforward in the field of healthcare where the principle of evidence-based practice is widely acknowledged and tools to change behaviour such as guidelines are often seen to be an implicit codification of evidence, despite the fact that this is not always the case [7]. Systematic reviews that explored changes in provider behaviour, but did not state that the changes were research based were excluded. Systematic reviews were excluded that made no mention of research evidence as were papers that were unclear about the use of research evidence [8,9]. Studies that focused on evidence-based interventions, but failed to report on the evidence base were also excluded. One systematic review was also excluded as it focused on changing patient rather than provider behaviour [10] and a second was excluded as it had no demonstrable outcomes [11].

## Results

Fifty-eight systematic reviews were read by members of the research team (either AF and AB or AF and JB) and 45 of these were excluded following discussion between all three members of the team in order to reduce the risk of bias. Thirty-one systematic reviews were excluded because they did not look explicitly at interventions designed to get research evidence into practice. Six systematic reviews were excluded because of unclear information about the evidence base of some individual studies within the review. In 5 cases it was clear that individual studies within the reviews focused on non-evidence-based changes such as cost reduction in prescribing practice and therefore these reviews were not exclusively based on getting research findings into practice. Three further papers were excluded as they were overviews. Additional file 2 provides bibliographic details and reasons for exclusion for the 45 excluded reviews.

We identified 13 systematic reviews that met the inclusion criteria. The systematic reviews contained a range of 10-66 primary studies. Of the 313 primary studies in the 13 systematic reviews, there were only 21 duplications. In the systematic reviews, Randomised Controlled Trials (RCT) were favoured by the authors over non-RCT study designs, however, non-randomised Controlled Clinical Trials (CCT), Before and After (B/A) and Interrupted Time Series (ITS) studies were also included. Several systematic reviews covered more than one clinical specialty while others focused on a specific area including prescribing, psychiatric care, pneumonia, obstetrics, stroke care and diabetes care. The original papers were all published in English, 5 came from Canada, 2 from Australia, 2 from the UK, one each from France, Germany, Italy and the USA.

The methodological quality of the systematic reviews was assessed by two members of the research team (either AF and AB or AF and JB) using an established quality checklist adapted by Franke et al from Oxman and Guyatt [5] using a scale of 0 (poor quality) to 7 (high quality). In most cases there was agreement between the two assessors. Where significant differences arose they were resolved by discussion between all three members of the review team. Nine of the systematic reviews received a maximum quality score i.e. 7 [12-20]. One systematic review received a score of 6 [21], two systematic reviews received a score of 5 [22,23] and one systematic review scored a 4 [24]. Further details are available in Additional file 3 in relation to each included systematic review. However, flaws identified within these systematic reviews related to: lack of clarity of search methods, lack of comprehensiveness of search methods, potential bias in the selection of articles and failure to report the methods used to combine the findings of the selected articles. The quality scores are listed in Table 1.

**Table 1:** Characteristics and results of included systematic reviews

Strategy type	Study reference	Systematic Review Quality score	Number of included studies	Conclusions: Effectiveness, Level of change, quality of individual studies
**Audit & Feedback**	**Bywood, P.T. et al ***Strategies for facilitating change in alcohol and other drugs (AOD) professional practice: a systematic review of the effectiveness of reminders and feedback *(2008)	5	14	**Effectiveness**: Reminders and feedback are effective strategies to facilitate professional practice change and have potential in the AOD field. **Level**: Small and/or non-significant changes in clients' health. **Quality**: Some risk of bias and/or other methodological flaws was evident in most studies

**Computerised decision support**	**Durieux, P. et al ***Computerized advice on drug dosage to improve prescribing practice (*2008)	7	26	**Effectiveness**: Some benefits, especially initial dosing. No effect on adverse reactions. **Level**: Small changes in process **Quality**: Although all studies used reliable outcome measures, their quality was generally low.

	**Mollon, B. et al ***Features predicting the success of computerized decision support for prescribing: a systematic review of randomized controlled trials *(2009)	7	41	**Effectiveness**: Potential exists to change health care provider behaviour; **Level**: Small changes in process, very few high quality studies show improvement in patient outcomes. **Quality**: Many studies poorly described

**Use of opinion leaders**	**Doumit, G. et al ***Local opinion leaders: effects on professional practice and health care outcomes (2007)*	7	12	**Effectiveness**: Can successfully promote Evidence Based Practice **Level**: Comparable with results for audit and feedback, education dissemination, and multifaceted interventions although smaller effect size than reminders. **Quality**: One study was judged to be of 'low risk'. Risk of bias in three studies was considered 'moderate'. Eight studies were judged to have 'high risk' of bias.

**Multifaceted interventions**	**Davey, P**. *Interventions to improve antibiotic prescribing practices for hospital inpatients *(2005)	7	66	**Effectiveness**: Interventions to improve antibiotic prescribing to hospital in-patients are successful, and can reduce antimicrobial resistance or hospital acquired infections. **Level**: Improved prescribing in at least one outcome measure for the majority of studies. **Quality**: The internal validity of the studies... is variable but there is a core of studies with low risk of bias or confounding'

	**Arnold, S. R**. *Interventions to improve antibiotic prescribing practices in ambulatory care*. (2005)	7	39	**Effectiveness**: The effectiveness of an intervention on antibiotic prescribing depends to a large degree on the particular prescribing behaviour and the barriers to change in the particular community. **Level**: Combined interventions resulted in moderate changes in prescribing behaviour and were more effective than single interventions, which resulted in small changes. **Quality**: Most of these studies had methodological limitations as assessed by the quality criteria of the EPOC study group

	**Weinmann, S. et al ***Effects of implementation of psychiatric guidelines on provider performance and patient outcome: systematic review *(2007)	6	18	**Effectiveness**: There is insufficient high-quality evidence to draw firm conclusions on the effects of implementation of specific psychiatric guidelines. **Level**: Mixed - but combined seem more effective than single interventions - the effects were moderate and temporary in most cases. **Quality**: Variable - overall a lack of high quality evidence hindered conclusions

	**Simpson, H. et al ***Do guidelines guide pneumonia practice? A systematic review of interventions and barriers to best practice in the management of community-acquired pneumonia *(2005)	4	6	**Effectiveness**: Combined interventions may be more successful than single interventions. **Level**: significant improvements in one or more measures of the process of pneumonia care. **Quality**: Variable

	**Harkennes, S. & Dodd, K**. *Guideline implementation in allied health professions: a systematic review of the literature *(2008)	7	14	**Effectiveness**: There is no evidence to support a set guideline implementation strategy for allied health professionals **Level**: Small to moderate effects detected. Results varied both within and between interventions. **Quality**: The methodological quality varied greatly

	**Chaillet, N. & Dumont, A**. *Evidence-based strategies for educing caesarean section rates: a meta-analysis *(2007)	7	10	**Effectiveness**: The caesarean section rate can be safely reduced by interventions that involve health workers in analyzing and modifying their practice **Level**: Combined more effective (especially when based on A&F) than single interventions. Identification of barriers to change is a key to success. **Quality**: All graded 'good' or 'fair' against EPOC guidelines

	**Chaillet, N. et al **E*vidence-based strategies for implementing guidelines in obstetrics *(2006)	7	33	**Effectiveness**: In the field of obstetric care, multifaceted strategy based on audit and feedback and facilitated by local opinion leaders is recommended to effectively change behaviours **Level**: Combined more effective (especially when based on A&F) than single interventions. **Quality**: All graded 'good' or 'fair' against EPOC guidelines

	**Kwan, J. et al ***Improving the efficiency of delivery of thrombolysis for acute stroke: a systematic review *(2004)	5	10	**Effectiveness**: Combined interventions may be more effective than single interventions **Level**: Small **Quality**: The description of study methodology and the intervention was generally satisfactory

	**De Belvis, A. G. et al ***Can primary care professionals' adherence to Evidence Based Medicine tools improve quality of care in Type 2 diabetes mellitus? A systematic review *(2009)	7	13	**Effectiveness**: The adherence to EBM instruments is likely to improve process of care, rather than patient outcomes. **Level**: Small **Quality**: Most of RCTs had methodological limitations

Table 1 shows the included studies with their quality scores, number of included studies and conclusions grouped by strategy types, which are drawn from EPOC implementation types. The authors of these reviews did not always report effect sizes and when they did they were descriptive (e.g. moderate or small) rather than numerical. The systematic reviews identify four strategy types; audit and feedback, computerised decision support, use of opinion leaders and multifaceted interventions that are considered in turn below. Multi-faceted interventions include more than one type of implementation strategy (including incentives, audit and feedback, educational strategies and reminders).

### Audit and feedback

One study looked at the effects of audit and feedback [22]. Prescribing and preventive care seem most likely to be altered by these approaches. More complex areas such as disease management, adherence to guidelines and diagnosis appear less effected by audit and feedback. The authors suggest that this may be due to the differences in complexity of the levels of decision making for clinicians in these respective facets of care.

### Computerised decision support

Two studies focused on computerised decision support. One review suggested that research evidence in the form of computer guidance may give clinicians greater confidence when prescribing and lead to more effective prescribing practice. A second review lamented the lack of high-quality primary studies demonstrating improvements in patient outcomes and the poor descriptive value of many studies which make learning lessons for implementation difficult [13]. However, the authors cautioned that the findings were based on a small number of studies and that the overall quality of these was low [12].

### Use of opinion leaders

One review looked at the role of local opinion leaders [14]. The authors suggest that opinion leaders can successfully promote evidence-based practice, however, the difficulty of identifying opinion leaders and the labour intensive nature of assessing their impact may limit the use of opinion leaders as a knowledge transfer intervention.

### Multifaceted interventions

The majority of the reviews incorporated studies that focused on more than one intervention type across a variety of clinical areas. Examples of the interventions in one multi-faceted approach included: physician and public education, physician peer review and incentive payments to physicians and hospitals. The most consistent message is that interventions designed to promote the use of evidence in policy are more effective when delivered as part of multifaceted intervention that combine different approaches [16,18-21,23,24], though the effect is characterised as small to moderate [17].

A further rationale for multifaceted interventions is that practitioners respond differently to varying types of interventions. For example, one of the reviews [18] investigated whether particular interventions were effective in promoting the use of evidence in obstetrics. They concluded that, in obstetrics, nurses were more receptive to educational strategies than physicians, whilst audit and feedback are effective for both groups.

Overall the reviews suggest that active interventions, such as opinion leaders [14] and reminders and feedback [22] are more effective than passive approaches, such as information campaigns.

## Discussion

This overview of systematic reviews, with its specific focus on evidence-based interventions, highlights a major limitation of existing reviews and primary studies in contributing to the effectiveness of Evidence-Based Medicine. This review emphasises the importance of ensuring that primary studies and systematic reviews are precise about the extent to which interventions are focused on changing practice based on evidence (as opposed to other information codified in guidelines, education material, etc.) The review identified very few systematic reviews looking exclusively and explicitly at implementing research findings into practice; conversely 43 reviews either focused on the implementation of non-evidenced based findings or were not explicit about the nature of the findings and were thus excluded.

This overview of systematic reviews updates the existing body of knowledge relating to the effectiveness of key mechanisms for improving clinical practice and service development [25,26]. The 13 studies included in this overview of systematic reviews highlights the small effects of single interventions such as audit and feedback, computerised decision support and opinion leaders. Multifaceted interventions are frequently used to promote the use of research in practice. Systematic reviews of multifaceted interventions claim an improvement in effectiveness over single interventions, with effect sizes ranging from small to moderate.

The EPOC group within the Cochrane Collaboration has made a particularly significant contribution in producing reviews relating to mechanisms such as audit and feedback [27], opinion leaders [14], and computerised advice [12]. Previous syntheses of existing reviews [1,4,28] have identified a large literature focused on changing practice, such as changing prescribing behaviour and service reorganizations. The literature focuses on a specific set of interventions that includes audit, clinical guidelines, opinion leaders and education and feedback. These interventions have been extensively evaluated in randomized controlled trials. The reviewers concluded that promoting the use of evidence in practice requires a complex, multifaceted intervention. While guidelines, feedback and educational interventions achieve small to moderate impacts in isolation, they are far more effective when combined in multiple strategies.

The challenges of achieving a more evidence-based approach to medical practice have been widely reported [29,30]. We have found that a number of published studies fail to state whether the recommended practice change is based on the best available research evidence. If this is not clearly stated in research papers it is not safe to assume this is the case. Furthermore, such an approach would run contrary to the principles of evidence-based medicine. Without being precise in this important matter we are in danger of assuming that all interventions designed to improve healthcare are implicitly evidence based, without research to support this hypothesis. Transparency and precision are critical to ensuring that evidence continues to play a key role in the development of healthcare and does not merely become shorthand for any 'desirable' change.

### Comparison with previous reviews

We know from the literature on the challenges involved in promoting Evidence-Based Medicine that the principles are not universally embedded in mechanisms such as guidelines and educational materials designed to promote clinical practice and service improvement [31]. It is therefore important that evaluations of strategies to change provider behaviour either only focus on changes that are evidenced based (not ones that are politically or financially driven) or are explicit about whether the changes are evidenced based or not.

In reporting the findings of existing primary studies, the systematic reviews point to two issues that warrant further investigation. Firstly, in order to improve the impact of research on health policy and practice, it is essential that theories are developed that reflect the diverse mechanisms involved in implementation [6]. It can be concluded from the reviews reported here that implementation of evidence into practice requires complex interventions that need to consider issues of context and process. For example, many of the systematic reviews [16,18-21,23,24] highlight the importance of multifaceted interventions to promote implementation of evidence into practice. One of the papers [18] signals the importance of considering what implementation mechanisms might be most effective in particular clinical contexts. Therefore, systematic reviews of effectiveness studies alone may not be sufficiently sensitive to deliver all the learning necessary to improve the use of research evidence in clinical practice and service improvement. A deeper understanding may be gained by complementing these studies with the findings from social science research that considers the important issues of context and process [32,33]. This review identified a much smaller literature focusing explicitly on getting research evidence into practice] [12-24]. This result suggests that further studies should explore whether the nature of the behaviour change being sought (either evidence based or not) has an impact on the degree of change that occurs.

However, the existence of a relatively large, rigorously evaluated set of interventions to promote the use of research evidence provides a vital tool (albeit not the only tool) in meeting the challenge of promoting better use of evidence in practice to improve patient care. Greater transparency and precision about the degree to which interventions are designed to promote evidence-based clinical practice and service improvement will further enhance our understanding of the progress made towards evidence-based medicine.

### Limitations and strengths of this study

There are some limitations to conducting overview reviews of systematic reviews. Firstly, there are concerns about double counting individual studies included in different reviews. In this overview we have checked for this and found surprisingly little overlap. Secondly, in reviews of reviews the studies identified are unlikely to have been published in the last few years, given the fact that they have been published in both an original paper and then identified and included in a published review. Thus a review of reviews is less likely to include the very latest research as this would not be captured in existing reviews. This might have particular implications for interventions based on new technologies such as electronic reminders for clinicians. We made best efforts to overcome this by running the searches again at the end of 2009 and incorporated 2 additional studies [13,20]. Finally, the reviewers are situated at some distance from the original studies and rely on summaries produced by others of existing primary studies. A further limitation related to the selection of systematic reviews that looked explicitly at interventions designed to get research evidence into practice. A number of systematic reviews were excluded as they were not explicit in their inclusion criteria that the studies selected were focused on promoting the use of evidence in practice. Others were excluded as they were not explicit in the main body of the text that the systematic review was focused on promoting the use of evidence in practice. These omissions may relate to reporting bias rather than the systematic reviews themselves.

However there is a considerable efficiency gain in doing a review of reviews, particularly as so much synthesis work has been done in the field already. We can learn from a wide body of work by reviewing 13 reviews of 313 individual studies. Furthermore, a coherent and tested set of interventions emerge that are highly consistent with previous studies [1].

## Ethical approval

Ethics committee approval was not required for this review.

## Declaration of Competing interests

The authors declare that they have no competing interests.

## Contributors

JB, AB and AF developed the review protocol, AF conducted the searches with guidance from Sarah Lawson (Senior Information Specialist NHS Support KCL) and conducted the initial screening based on titles and abstracts. Full text screening was conducted by JB, AB and AF. Data extraction and quality appraisal was conducted by either JB and AF or AB and AF. The first draft of the paper was produced by AB and JB, with subsequent drafts developed by AB, JB and AF. All authors have read and approved the manuscript.

## Supplementary Material

Additional file 1 **Data extracted from the included reviews**. Detailed information about the included reviews and reasons for inclusion. Click here for file

Additional File 2 **Excluded studies**. Information about the studies excluded from the review. Click here for file

Additional file 3 **Quality assessment**. A description of the quality assessment tools used in the review. Click here for file
