# Supplementary material for: Effective implementation of research into practice: an overview of systematic reviews of the health literature
Source: BMC Res Notes. 2011 Jun 22;4:212. doi: 10.1186/1756-0500-4-212 (PMC3148986; doi:10.1186/1756-0500-4-212)
Supplement: Additional file 1 — Data extracted from the included reviews. Detailed information about the included reviews and reasons for inclusion. [file 1756-0500-4-212-S1.DOC]

**Additional file 1**

**Title: Data extracted from the included reviews**.

Description: Detailed information about the included reviews and reasons for inclusion

| **Column 1**  **References, aims and short description** | **Column 2**  **Methodological characteristics** | **Column 3**  **Results and conclusions** | **Column 4**  **Reasons for inclusion** |
| --- | --- | --- | --- |
| **Arnold, S. R. *Interventions to improve antibiotic prescribing practices in ambulatory care.* (2007)**  **Aim:** To estimate the effectiveness of professional interventions, alone or in combination, in improving the selection, dose and treatment duration of antibiotics prescribed by healthcare providers in the outpatient setting; and to evaluate the impact of these interventions on reducing the incidence of antimicrobial resistant pathogens.  **Studies included:** 16 N. America, 5 UK, 5 Australia/New Zealand, 3 Norway, 2 Spain, 1, Sri Lanka, 1 Zambia, 1, Sweden, 1 S. Africa, 1 Mexico, 1 Indonesia, 1 Finland, 1 Netherlands.  **Clinical area of interest:** Prescribing in an OP setting  **Participants:** Studies of healthcare consumers, qualified physicians of all ages and level of experience and physician extenders who prescribe antibiotics and provide primary care in community or academic ambulatory settings were included. Studies including only medical trainees were excluded  **Intervention(s):** Distribution of educational materials, educational meetings, local consensus processes, EOV, local opinion leaders, patient mediated interventions, A&F, reminders, marketing, mass media, financial interventions. | **Design of studies included:** RCT, QRCT, CBA, ITS  **Time frame:** 1966-2000  **Quality score:** 7  **Quality of studies included:** *‘Most of these studies had methodological limitations as assessed by the quality criteria of the EPOC study group’* (p7) | **Characteristics of implementation strategies:**  These interventions addressed the overuse of antibiotics for viral infections. Use of printed educational materials or audit and feedback alone resulted in no or only small changes in prescribing. The exception was a study documenting a sustained reduction in macrolide use in Finland following the publication of a warning against their use for group A streptococcal infections. Interactive educational meetings appeared to be more effective than didactic lectures.  Educational outreach visits and physician reminders produced mixed results. Patient-based interventions, particularly the use of delayed prescriptions for infections for which antibiotics were not immediately indicated effectively reduced antibiotic use by patients and did not result in excess morbidity. Multi-faceted interventions combining physician, patient and public education in a variety of venues and formats were the most successful in reducing antibiotic prescribing for inappropriate indications. Only one of four studies demonstrated a sustained reduction in the incidence of antibiotic-resistant bacteria associated with the intervention.  **Authors conclusions:**  The effectiveness of an intervention on antibiotic prescribing depends to a large degree on the particular prescribing behaviour and the barriers to change in the particular community. No single intervention can be recommended for all behaviours in any setting. Multi-faceted interventions where educational interventions occur on many levels may be successfully applied to communities after  addressing local barriers to change. | **Reasons for inclusion:** Focus is on the implementation of research evidence into practice  Authors caution against over-generalising from their findings, however, this review suggests that simple, single-intervention studies generally have a small impact on behaviour. The authors speculate that this may be because the reasons providers behave as they do are too ingrained and multi-factoral to be changed by singular interventions. |

| **Column 1**  **References, aims and short description** | **Column 2**  **Methodological characteristics** | **Column 3**  **Results and conclusions** | **Column 4**  **Reasons for inclusion** |
| --- | --- | --- | --- |
| **Bywood, P.T. et al *Strategies for facilitating change in alcohol and other drugs (AOD) professional practice: a systematic review of the effectiveness of reminders and feedback* (2008)**  **Aim:** Evaluation of the effectiveness of reminders and feedback in changing professional practice  **Studies included:** Locations unspecified – English language only  **Clinical area of interest:** Addiction (alcohol and other drugs) Studies included from a variety of other (general health care) areas however.  **Participants:** Practitioners, clients  **Intervention(s):** Reminders, feedback | **Design of studies included:** Controlled studies  **Time frame:** 1966-March 2005 (various databases  **Quality score:** 5  **Quality of studies included:** *‘Some risk of bias and/or other methodological flaws was evident in most studies, including those evaluated previously in existing reviews’* (p550) | **Characteristics of implementation strategies:**  Fourteen existing systematic reviews and 15 primary studies were assessed. Because few studies evaluated the effectiveness of  reminders and feedback in the AOD context, evidence is drawn largely from the general health-care literature. Use of reminders and  feedback is supported for a range of health behaviours. AOD specific clinical behaviours that are most likely to be improved with  the use of reminders or feedback include pharmacotherapy prescribing, AOD education, screening and counselling and monitoring/ management of AOD treatment and/or related problems (e.g. depression).  **Authors conclusions:**  Reminders and feedback are effective strategies to facilitate professional practice change and have potential in the AOD field. However, further well-designed empirical studies are needed to assess fully the effectiveness of these professional practice change strategies in AOD-specific contexts. | **Reason for inclusion:** Focus is on the implementation of research evidence into practice  Prescribing and preventive care seem most likely to be altered by these approaches – more complex areas such as disease management, adherence to guidelines and diagnosis appear less so - the authors suggest this may be due to the differences here of clinician complexity levels in decision making. Once again there is a lack of evidence relating to outcomes |

| **Column 1**  **References, aims and short description** | **Column 2**  **Methodological characteristics** | **Column 3**  **Results and conclusions** | **Column 4**  **Reasons for inclusion** |
| --- | --- | --- | --- |
| **Chaillet, N. et al *Evidence-based strategies for implementing guidelines in obstetrics* (2006)**  **Aim:** Greater understanding of guideline implementation  **Studies included:** International (places of studies not comprehensively listed)  **Clinical area of interest:** Obstetrics  **Participants:** (Obstetric) healthcare providers  **Intervention(s):** Educational strategies with medical providers and paramedical providers, use of opinion leaders, qualitative improvement, academic detailing, A&F, reminders and multifaceted strategies. | **Design of studies included:** RCT, CBA, ITS  **Time frame:** January 1990-June 2005  **Quality score:** 7  **Quality of studies included:** All graded ‘good’ or ‘fair’ against EPOC guidelines | **Characteristics of implementation strategies:**  The minimum inclusion criteria were objective measurement of performance, relevant and interpretable data present or obtainable, contemporary data collection and appropriate choice of control site (for controlled before-after studies), intervention time clearly defined, and at least three data points before and three after the intervention (for interrupted time series studies). Interrupted time series studies with only one published data point before and three after were also included if authors provided information about regional secular trend for the first outcome.  Educational strategies ineffective in changing physicians behaviour (mixed effect with paramedical public) Opinion leader, quality improvement, and academic detailing have mixed effects. Audit and feedback, reminders and multi-faceted strategies generally effective  **Authors conclusions:**  Prospective identification of efficient strategies and barriers to change is necessary to achieve a better adaptation of intervention and to improve clinical practice guidelines implementation. In the field of obstetric care, multifaceted strategy based on audit and feedback and facilitated by local opinion leaders is recommended to effectively change behaviours. | **Reason for inclusion:** Focus is on the implementation of research evidence into practice  This study is useful in suggesting there is a difference in ‘what works’ in implementation in obstetrics to other fields – this may be in part to do with the obstetric environment (including, for e.g. medical legal concerns)  Variety of approaches considered is comprehensive, authors caution there may be a publication bias (23 studies effective/mixed results versus 6 ineffective) therefore review findings must be considered with caution. |

| **Column 1**  **References, aims and short description** | **Column 2**  **Methodological characteristics** | **Column 3**  **Results and conclusions** | **Column 4**  **Reasons for inclusion** |
| --- | --- | --- | --- |
| **Chaillet, N. & Dumont, A. *Evidence-based strategies for educing caesarean section rates: a meta-analysis* (2007)**  **Aim:** To assess the effectiveness of interventions for reducing the caesarean section rate and to assess the impact of this reduction on maternal and perinatal mortality and morbidity  **Studies included:** 6 USA, 1 S. America, 1 Australia, 1 Taiwan, 1 UK  **Clinical area of interest:** Obstetrics  **Participants:** Healthcare providers  **Intervention(s):** A&F, Quality improvement, multifaceted strategies | **Design of studies included:** RCT, quasi RCT, controlled BA, ITS  **Time frame:** January 1990-June 2005  **Quality score:** 7  **Quality of studies included:** All graded ‘good’ or ‘fair’ against EPOC guidelines | **Characteristics of implementation strategies:**  Among the 10 included studies, a significant reduction of caesarean  section rate was found by random meta-analysis (pooled RR=0.81 [0.75, 0.87]). No evidence of publication bias was identified. Audit and feedback (pooled RR = 0.87 [0.81, 0.93]), quality improvement (pooled RR=0.74 [0.70, 0.77]), and multifaceted strategies (pooled RR=0.73 [0.68, 0.79])were effective for reducing the caesarean section rate .However, quality improvement based on active management of labor showed mixed effects. Design of studies showed a higher effect for non-controlled studies than for controlled studies (pooled RR = 0.76 [0.72, 0.81] vs 0.92 [0.88, 0.96]). Studies including an identification of barriers to change were more effective than other interventions for reducing the caesarean section rate (pooled RR=0.74 [0.71, 0.78] vs 0.88 [0.82, 0.94]). Among included studies, no significant differences were found for perinatal and neonatal mortality and perinatal and maternal morbidity with respect to the mode of delivery. Only 1 study showed a significant reduction of neonatal and perinatal mortality (p<0.001).  **Authors conclusions:**  The caesarean section rate can be safely reduced by interventions that involve health workers in analyzing and modifying their practice. Our results suggest that multifaceted strategies, based on audit and  detailed feedback, are advised to improve clinical practice and effectively reduce caesarean section rates. Moreover, these findings support the assumption that identification of barriers to change is a major key to success. | **Reason for inclusion:** Focus is on the implementation of research evidence into practice  Useful study pointing to barriers/facilitators to change – external changes, practice environment – ie unit leadership, policy, equipment availability, potential adopters, strategies to promote the uptake of guideline recommendations. The suggestion is that studies which identified these key factors were more successful than those that did not – contextual understanding seems important as does a multifaceted approach to behavioural change. |

| **Column 1**  **References, aims and short description** | **Column 2**  **Methodological characteristics** | **Column 3**  **Results and conclusions** | **Column 4**  **Reasons for inclusion** |
| --- | --- | --- | --- |
| **Davey, P. *Interventions to improve antibiotic prescribing practices for hospital inpatients* (2005)**  **Aim:** To estimate the effectiveness of professional interventions that alone, or in combination, are effective in promoting prudent antibiotic prescribing to hospital inpatients  **Studies included:** 42 USA, 2 Australia, 1 Brazil, 4 Canada, 1 Columbia, 2 France, 2 Netherlands, 1 Norway, 1 Spain, 2 Thailand, 8 UK  **Clinical area of interest:** Prescribing IP setting  **Participants:** Health care professionals who prescribe antibiotics to hospital in-patients receiving acute care  **Intervention(s):** 1) Persuasive interventions: distribution of educational materials; educational meetings; local consensus processes; educational outreach visits; local opinion leaders; reminders provided verbally, on paper or on computer; audit and feedback.  (2) Restrictive interventions. These include selective reporting of laboratory susceptibilities, formulary restriction, requiring prior authorisation of prescriptions by infectious diseases physicians, microbiologists, pharmacists etc., therapeutic substitutions, automatic stop orders and antibiotic policy change strategies including cycling, rotation and crossover studies.  (3) Structural: the influence on antibiotic prescribing of changing from paper to computerised records and of the introduction or organisation of quality monitoring mechanisms. | **Design of studies included:** RCT, CCT, CBA, ITS  **Time frame:** 1966-2003  **Quality score:** 7  **Quality of studies included:** ‘*The internal validity of the studies..is variable but there is a core of studies with low risk of bias or confounding’* (p43) | **Characteristics of implementation strategies:**  Sixty-six studies were included and 51 (77%) showed a significant improvement in at least one outcome. Six interventions only aimed to increase treatment, 57 interventions aimed to decrease treatment and three interventions aimed to both increase and decrease treatment. The intervention target was the decision to prescribe antibiotics (one study), timing of first dose (six studies), the regimen (drug, dosing interval etc, 61 studies) or the duration of treatment (10 studies); 12 studies had more than one target. Of the six interventions that aimed to increase treatment, five reported a significant improvement in drug outcomes and one a significant improvement in clinical outcome. Of the 60 interventions that aimed to decrease treatment, 47 reported drug outcomes of which 38 (81%) significantly improved, 16 reported microbiological outcomes of which 12 (75%) significantly improved and nine reported clinical outcomes of which two (22%) significantly deteriorated and 3 (33%) significantly improved. Five studies aimed to reduce CDAD. Three showed a significant reduction in CDAD.  **Authors conclusions:**  The results show that interventions to improve antibiotic prescribing to hospital in-patients are successful, and can reduce antimicrobial resistance or hospital acquired infections. | **Reason for inclusion:** Focus is on the implementation of research evidence into practice  High number of different interventions surveyed in a high number of countries. Findings suggest provider behaviour can be changed – however authors suggest that these 66 studies represent only 20% of the literature here – many studies could not be included due to methodological flaws. Review suggests that ITS studies are more useful than RCT/CCT as ITS studies show pre-intervention baseline and degree of sustainability of intervention. Two other interesting conclusions relating to behaviour change and practice; firstly that interventions are less likely to be successful if there is evidence that practice is already changing in the desired direction. Secondly, and possibly more importantly, restrictive interventions have a greater immediate impact than persuasive interventions. The authors stress this is a tentative finding – however this distinction may be useful. |

| **Column 1**  **References, aims and short description** | **Column 2**  **Methodological characteristics** | **Column 3**  **Results and conclusions** | **Column 4**  **Reasons for inclusion** |
| --- | --- | --- | --- |
| **De Belvis, A. G. et al** ***Can primary care professionals’ adherence to Evidence Based***  ***Medicine tools improve quality of care in Type 2 diabetes mellitus? A systematic review* (2009)**  **Aim:** To review the effectiveness of EBM tools available to primary care professionals to improve the quality of Type 2 diabetes disease management  **Studies included:** Not explicit re countries  **Clinical area of interest:** Diabetes  **Participants:** Primary health care providers (physicians and nurses).  **Intervention(s):** Practice guidelines, recommendations, clinical and/or integrated care pathways, audit, disease management programme, ICT devices, training | **Design of studies included:** RCTs  **Time frame:** 1988-2009  **Quality score:** 7  **Quality of studies included:** *‘Most of RCTs had methodological limitations’* (p125) | **Characteristics of implementation strategies:**  13 RCTs included in the analysis.  (1) educational training, including face-to-face training individual or grouped sections, manuals for self-directed learning, patient management flow-charts, practice based education and newsletters  (2) internal or external audit, including feedback reports on performance and peer review;  (3) ICT devices, such as computer-based reminders, phone-call reminders and SMS reminders  (4) combination among the different kind of interventions.  As for the proposals, University and Scientific Institutions proposed 4 interventions; Health Care Organizations 7; Private insurances and/or HMOs 1.  **Authors conclusions:**  The adherence to EBM instruments is likely to improve process of care, rather than patient outcomes. In addition, our review outlines that feedback reports and use of ICT devices are likely to be effective in diabetes disease management. | **Reason for inclusion:**  Focus is on the implementation of research evidence into practice  Strong focus on methods to increase uptake of EBM in to practice. Dual role of ICT interventions – useful in increasing guideline uptake by providers and can be used to audit performance. Process more likely to be improved than outcomes – though only one study measured both. Small number of studies – black box, Hawthorne effects noted by authors, and short timescale of F/U in studies noted by authors mean we must interpret these results with caution. |

| **Column 1**  **References, aims and short description** | **Column 2**  **Methodological characteristics** | **Column 3**  **Results and conclusions** | **Column 4**  **Reasons for inclusion** |
| --- | --- | --- | --- |
| **Doumit, G. et al *Local opinion leaders: effects on professional practice and health care outcomes (2007)***  **Aim:** To assess the effectiveness of the use of local opinion leaders in improving the behaviour of health care professionals and patient outcomes  **Studies included:** 9 USA, 2 Canada, 1 China  **Clinical area of interest:** IP & OP settings (various specialities) including O&G, Cardiology, Oncology, COPD  **Participants:** Healthcare professionals in charge of patient care.  **Intervention(s):** Opinion leaders. | **Design of studies included:** RCTs only  **Time frame:** 1966-Feb 2005  **Quality score:** 7  **Quality of studies included:** *‘One study was judged to be of ‘low risk’... risk of bias in three studies was considered ‘moderate’... eight studies were judged to have ‘high risk’ of bias’*(p16)  **Inclusion: ‘Opinion leaders promote evidence based practice’** | **Characteristics of implementation strategies:**  12 studies met the authors’ criteria.  Authors defined local opinion leaders as those that are identified by one the following methods:  (i) Sociometric method  (ii) Informant method  (iii) Self designating method  (iv) Observation method.  Studies that did not utilise any of the above methods were excluded.  The adjusted absolute risk difference of non-compliance with desired practice varied from - 6% (favouring control) to +25% (favouring opinion leader intervention). Overall, the median adjusted risk difference (ARD) was 0.10 representing a 10% absolute decrease in non-compliance in the intervention group.  **Authors conclusions:**  The use of local opinion leaders can successfully promote evidence-based practice. However the feasibility of its widespread use remains uncertain. | **Reason for inclusion:**  Focus is on the implementation of research evidence into practice  Sociometric method was most common way of identifying opinion leaders. The evidence here does not confirm Ryan’s (2002) hypothesis that formalising opinion leaders diminishes their influence. Limits of this study include lack of agreed definition of what an opinion leader may or may not be across the 12 studies – therefore it is difficult to posit what makes an opinion leader more or less successful. Authors put this study in the context of other studies to conclude that opinion leaders appear comparable to the distribution of educational materials, A&F, and multi-faceted interventions in reducing non-compliance with desired practice. However, the effect sizes reported here appear smaller than those associated with reminder systems. Finally the difficulty of identifying opinion leaders and the labour intensive nature of assessing their impact may limit the use of opinion leaders as a knowledge transfer intervention. |

| **Column 1**  **References, aims and short description** | **Column 2**  **Methodological characteristics** | **Column 3**  **Results and conclusions** | **Column 4**  **Reasons for inclusion** |
| --- | --- | --- | --- |
| **Durieux P et al *Computerized advice on drug dosage to improve prescribing practice (*2008)**  **Aim:** Examination of whether computerised advice on drug dosage has beneficial effects on the process or outcome of health care  **Studies included:** 12 USA, 1 Canada, 1 Australia, 2 New Zealand, 1 Isreal, 9 Europe (UK, France, Spain, Italy)  **Clinical area of interest:** Prescribing in IP & OP settings  **Participants:** Health professionals responsible for patient care.  **Intervention(s):** All comparative studies of computer advice on drug dosage | **Design of studies included:** RCT, CCT, BA, ITS  **Time frame:** EPOC 1996-December 2006, Medline 1966-December 2006, EMBASE 1980-December 2006, Therapeutic Drug Monitoring 1979-March 2007, JAMIA 1996-March 2007  **Quality score:** 7  **Quality of studies included: *‘****Although all studies used reliable outcome measures, their quality was generally low’* | **Characteristics of implementation strategies:**  Twenty-six comparisons (23 articles) were included (as compared to fifteen comparisons in the original review) including a wide range  of drugs in inpatient and outpatient settings. Interventions usually targeted doctors although some studies attempted to influence  prescriptions by pharmacists and nurses. Although all studies used reliable outcome measures, their quality was generally low.  **Authors conclusions:**  Computerized advice for drug dosage has some benefits: it increased the initial dose of drug, increased serum drug concentrations and led to a more rapid therapeutic control. It also reduced the risk of toxic drug levels and the length of time spent in the hospital.  However, it had no effect on adverse reactions. In addition, there was no evidence to suggest that some decision support technical features (such as its integration into a computer physician order entry system) or aspects of organization of care (such as the setting) could optimise the effect of computerised advice. | **Reason for inclusion:** Focus is on the implementation of research evidence into practice  Effectively, research evidence in the form of computer guidance may give clinicians greater confidence when prescribing – hence prescribing in a more effective (and less conservative) way. Thus the evidence base upon which prescribing decisions are made may be more robust.  However, the authors stress that we need to read these results with caution for the following reasons: 1, the findings are based on a small number of studies focussed on a small number of drugs. 2, the quality of studies was generally low. 3, heterogeneity across individual comparisons was high for most outcomes. 4, Indicators such as length of stay/mortality can be crude – they may be affected by other factors. |

| **Column 1**  **References, aims and short description** | **Column 2**  **Methodological characteristics** | **Column 3**  **Results and conclusions** | **Column 4**  **Reasons for inclusion** |
| --- | --- | --- | --- |
| **Harkennes, S. & Dodd, K. *Guideline implementation in allied health professions: a systematic review of the literature* (2008)**  **Aim:** To evaluate the effects of the introduction of clinical guidelines for allied health professionals, and to estimate the effectiveness of the guideline dissemination and implementation strategies used.  **Studies included:** 8 USA, 2 Australia, 2 Netherlands, 2 UK  **Clinical area of interest:** AHPs guideline adherence - AHPs. For the purposes of this review, allied health included the following professions: audiology; dietetics; occupational therapy; orthoptics; orthotics and prosthetics; pharmacy; physiotherapy; podiatry; psychology; radiography; speech pathology; and social work.  **Participants:** AHPs  **Intervention(s):** Clinical guidelines were defined as ‘‘systematically developed statements to assist practitioner and patient decisions about appropriate health care for specific clinical circumstances’’. All types of dissemination and implementation strategies were included. | **Design of studies included:** RCT, CCT, controlled B/A, ITS  **Time frame:** 1966-June 2006 (various databases)  **Quality score:** 7  **Quality of studies included:** Fourteen studies met the inclusion criteria. The methodological quality varied greatly, with the proportion of quality criteria met ranging from 0 to 6 out of seven. | **Characteristics of implementation strategies:**  Studies were included if they reported objective measures of change in the allied health practitioners’ behaviour or patient outcomes. Studies measuring other outcomes, such as change in knowledge or attitudes of the allied health practitioner and costs were only included if change in practitioner behaviour or patient outcome were also measured. In most studies, the effects reported for patient and process outcomes were small and in favour of the intervention group. Of the 14 included studies, 10 focused on educational interventions. Six of the 14 studies used a single intervention strategy and seven used a multifaceted implementation strategy. One study compared both single and multi-faceted strategies. Multi-faceted interventions were no more effective than single intervention strategies and effects of the same strategy varied across trials.  **Authors conclusions:**  There is no evidence to support a set guideline implementation strategy for allied health professionals. When implementing clinical guidelines it is important to first identify specific barriers to change using theoretical frameworks of behaviour change and then develop strategies that deal with these barriers. When measuring the effectiveness of these strategies, professionals should consider the use of both patient and process outcomes and choose outcomes that reflect their aims | **Reason for inclusion:** Focus is on the implementation of research evidence into practice  Interesting conclusion re difficulty of generalising/standardising approaches from above – rather the applicability of a successful framework may come from below – does this point towards an action research approach? Context seems key once more. Useful to see this from an AHP perspective – how does this compare with other professional groups’ experiences? |

| **Column 1**  **References, aims and short description** | **Column 2**  **Methodological characteristics** | **Column 3**  **Results and conclusions** | **Column 4**  **Reasons for inclusion** |
| --- | --- | --- | --- |
| **Kwan, J. et al *Improving the efficiency of delivery of thrombolysis for acute stroke: a systematic review* (2004)**  **Aim:** Interventions to overcome barriers to rapid administration of thrombolytic therapy  **Studies included:** 6 USA, 2 Canada, 1 Germany, 1 UK  **Clinical area of interest:** Stroke care  **Participants:** Public, paramedics, ED & IP healthcare providers  **Intervention(s):** Education for public, training programme for paramedics, helicopter transfer of stroke patients, training programme for ED staff, re-organisation of in-hospital system | **Design of studies included:** Non-randomised clinical study, BA, observational studies  **Time frame:** Up to 2002  **Quality score:** 5  **Quality of studies included:** *‘The description of study methodology and the intervention was generally satisfactory’* (p274) | **Characteristics of implementation strategies:**  10 studies included. Public and staff educational campaigns appeared to have some effect in reducing the delay to receiving thrombolysis. Training of paramedical staff could improve the speed of hospital admission. Use of helicopters produced mixed results. Re-organisation of in-hospital systems (2 studies) one showed all-round reduction of delays, the other was less effective in reducing delays overall. Multifaceted studies focussing on ED and stroke unit staff and protocols seemed to have overall impressive results  **Authors conclusions:**  Multifaceted programmes might be more likely to be successful in reducing delays to therapy | **Reason for inclusion:** Focus is on the implementation of research evidence into practice  Highlights the complex nature of stroke care and suggests ways that the evidence based knowledge of how to minimise stroke effects may be maximised – i.e. increase public knowledge of what stroke is, reduce delays in getting to hospital, reduce delays once in hospital, and ensure the most appropriate healthcare provider assesses and treats the patient as soon as possible. |

| **Column 1**  **References, aims and short description** | **Column 2**  **Methodological characteristics** | **Column 3**  **Results and conclusions** | **Column 4**  **Reasons for inclusion** |
| --- | --- | --- | --- |
| **Mollon, B. et al *Features predicting the success of computerized decision support for prescribing: a systematic review of randomized controlled trials* (2009)**  **Aim: 1)** When evaluated rigorously in randomized controlled trials, have current RxCDSS successfully been implemented and altered physician prescribing or patient outcomes? Furthermore, (2) what features of these RxCDSS are associated with success versus failure?  **Studies included:** Not explicit re countries  (all English language)  **Clinical area of interest:** Twenty-three studies (56.1%) used a RxCDSS in an outpatient general practice or internal medicine setting, 10 (24.4%) in inpatient hospital wards or emergency rooms, 5 (12.2%) in pharmacies and 3 (7.3%) in specialty clinics (2 for paediatrics and 1 for diabetes). The systems addressed a variety of problems – cardiovascular care (36.6%), general/internal medicine (29.3%), diabetes (9.8%), respiratory disease (9.8%), otitis media (7.3%), depression, osteoporosis and infectious disease (2.4% each). Nineteen (46.3%) of the RxCDSS were integrated with drug order entry, 16 (39.0%) with management/electronic health record (EHR) software and 9 (22.0%) also printed the suggestions  **Participants:** Primarily physicians  **Intervention(s):** An intervention which utilized a computer to analyze patient-specific information to advise a prescriber (primarily a physician) or pharmacist when they were writing or filling a prescription, respectively | **Design of studies included:** RCTs  **Time frame:** Up to June 2008  **Quality score:** 7  **Quality of studies included:** variable – robustly analysed by authors | **Characteristics of implementation strategies:**  Of 4534 citations returned by the search, 41 met the inclusion criteria. Of these, 37 reported successful system implementations, 25 reported success at changing health care provider behaviour, and 5 noted improvements in patient outcomes. A mean of 17 features per study were mentioned. The statistical analysis could not be completed due primarily to the small number of studies and lack of diversity of outcomes. Descriptive analysis did not confirm any feature to be more prevalent in successful trials relative to unsuccessful ones for implementation, provider behaviour or patient outcomes.  **Authors conclusions:**  While RxCDSSs have the potential to change health care provider behaviour, very few high quality studies show improvement in patient outcomes. Furthermore, the features of the RxCDSS associated with success (or failure) are poorly described, thus making it difficult for system design and implementation to improve. | **Reason for inclusion:**  Focus is on the implementation of research evidence into practice  Focus is on reduction of prescribing errors and making prescribing more evidence based. |

| **Column 1**  **References, aims and short description** | **Column 2**  **Methodological characteristics** | **Column 3**  **Results and conclusions** | **Column 4**  **Reasons for inclusion** |
| --- | --- | --- | --- |
| **Simpson, H. et al *Do guidelines guide pneumonia practice? A systematic review of interventions and barriers to best practice in the management of community-acquired pneumonia* (2005)**  **Aim:** To evaluate the effects of guidelines in CAP care  **Studies included:** Not explicit re countries of origin – English language only.  **Clinical area of interest:** CAP  **Participants:** Healthcare providers  **Intervention(s):** Guidelines, implementation strategy, clinical pathway | **Design of studies included:** RCT, ITS, controlled BA  **Time frame:** 1966-July 2004  **Quality score:** 4  **Quality of studies included:** Variable. Of note; 6 primary studies results presented alongside findings of 8 other studies focussing on ‘barriers’ to guideline implementation. | **Characteristics of implementation strategies:**  6 studies evaluated the effectiveness of a guideline-based intervention with adequate scientific rigor. Of these 6 studies, 3 showed a significant reduction in length of stay when treatment of CAP followed guideline recommendations. All six studies also reported significant improvements in one or more measures of the process of pneumonia care.  **Authors conclusions:**  Successful guideline implementation programs need to understand local barriers, incorporate multiple component interventions, and proceed within a framework of continuous quality improvement. | **Reason for inclusion:** Focus is on the implementation of research evidence into practice  This study evaluates methods of implementing EB guidelines into practice. Useful section on barriers to adoption and implementation – authors suggest that the voluntary nature of guidelines mean that physicians may choose to ignore them. Likewise patient age is a factor – the older the patient, the less likely guidelines are to be applied. There are also contextual systemic factors which impede the uptake of guidelines. The theme of micro/meso factors and the need for alignment across levels is raised. |

| **Column 1**  **References, aims and short description** | **Column 2**  **Methodological characteristics** | **Column 3**  **Results and conclusions** | **Column 4**  **Reasons for inclusion** |
| --- | --- | --- | --- |
| **Weinmann, S. et al *Effects of implementation of psychiatric guidelines on provider performance and patient outcome: systematic review* (2007)**  **Aim:** Examination of guideline implementation – greater understanding  **Studies included:** 12 USA, 4 UK, 1 Denmark, 1 Canada  **Clinical area of interest:** Psychiatry  **Participants:** Psychiatrists, GPs, patients  **Intervention(s):** The authors defined guidelines as systematically developed statements to assist practitioner decisions about appropriate health care in specific clinical circumstances. | **Design of studies included:** RCT, CCT, BA  **Time frame:** 1966-March 2006 (English, German, French, Italian, Spanish language)  **Quality score:** 6  **Quality of studies included:** Variable – overall a lack of high quality evidence hindered conclusions | **Characteristics of implementation strategies:**  Methods used: CQI, academic detailing, distribution of academic materials, marketing techniques, A&F, patient mediated interventions, reminders. 18 Studies included (nine randomized-controlled trials, six nonrandomized- controlled studies and three quasi-experimental before-and-after studies) were identified. Effects on provider performance or patient outcome were moderate and temporary in most cases. Studies with positive outcomes used complex multifaceted interventions or specific psychological methods to implement guidelines.  **Authors conclusions:**  There is insufficient high-quality evidence to draw firm conclusions on the effects of implementation of specific psychiatric guidelines. | **Reason for inclusion:** Focus is on the implementation of research evidence into practice Authors are sceptical that changing clinician performance and improving guideline adherence rates will necessarily lead to better outcomes – overall their findings are equivocal – they point to a study that in fact suggests greater guideline adherence leads to increased medication usage, and increased medication side-effects. Is this due to the nature of psychiatry, or these specific studies/techniques, or can these findings be extrapolated to other clinical settings? |

**Key: RCT, randomized controlled trial; QRCT, quasi-randomized controlled trial; CCT, controlled clinical trial; CA, controlled after; UA, uncontrolled after; BA, before/after; XS, cross sectional; ITS, interrupted time series.**
