# Supplementary material for: Effective implementation of research into practice: an overview of systematic reviews of the health literature
Source: BMC Res Notes. 2011 Jun 22;4:212. doi: 10.1186/1756-0500-4-212 (PMC3148986; doi:10.1186/1756-0500-4-212)
Supplement: Additional File 2 — Excluded studies. Information about the studies excluded from the review. [file 1756-0500-4-212-S2.DOCX]

**Additional File 2**

**Title: Excluded studies**

**Description: Information about the studies excluded from the review**

| **Author** | **Jamtvedt G, Young JM, Kristoffersen DT, O’Brien MA, Oxman AD.** |
| --- | --- |
| **Title** | **Audit and feedback: effects on professional practice and health care**  **outcomes. *Cochrane Database of Systematic Reviews*** |
| **Year** | 2006 |
| **Reason for exclusion** | The link between research evidence and practice is not sufficiently explicit. |

| **Author** | **O’Brien MA, Rogers S, Jamtvedt G, Oxman AD, Odgaard-Jensen J, Kristoffersen DT, Forsetlund L, BainbridgeD, FreemantleN, DavisD, HaynesRB, Harvey E.** |
| --- | --- |
| **Title** | **Educational outreach visits: effects on professional practice and health care outcomes. *Cochrane Database of Systematic Reviews* 2007** |
| **Year** | 2007 (update of 1997 study) |
| **Reason for exclusion** | A significant number of studies encouraging the uptake of drugs based on financial considerations are included in this systematic review |

| **Author** | **Shiffman, R. N. et al** |
| --- | --- |
| **Title** | **Computer-based guideline implementation systems: a systematic review of functionality and effectiveness** |
| **Year** | 1999 |
| **Reason for exclusion** | A significant number of studies encouraging the uptake of drugs based on financial considerations are included in this systematic review |

| **Author** | **Chaix-Couturier. C, et al** |
| --- | --- |
| **Title** | **Effects of financial incentives on medical practice: results from a systematic review of the literature and methodological issues** |
| **Year** | 2000 |
| **Reason for exclusion** | The link between research evidence and practice is not sufficiently explicit. |

| **Author** | **Gill, P. S. et al** |
| --- | --- |
| **Title** | **Changing doctor prescribing behaviour** |
| **Year** | 1999 |
| **Reason for exclusion** | Unclear information about the evidence base of some individual studies within the review |

| **Author** | **Pearson, S. A. et al** |
| --- | --- |
| **Title** | **Changing Medication Use in Managed Care: A Critical Review of the Available Evidence** |
| **Year** | 2003 |
| **Reason for exclusion** | The link between research evidence and practice is not sufficiently explicit. |

| **Author** | **Von Gunten, V. et al** |
| --- | --- |
| **Title** | **Clinical and economic outcomes of pharmaceutical services related to antibiotic use: a literature review** |
| **Year** | 2007 |
| **Reason for exclusion** | A significant number of studies encouraging the uptake of drugs based on financial considerations are included in this systematic review |

| **Author** | **Bennett, J. W. And Glasziou, P. P.** |
| --- | --- |
| **Title** | **Computerised reminders and feedback in medication management:**  **a systematic review of randomised controlled trials** |
| **Year** | 2003 |
| **Reason for exclusion** | A significant number of studies encouraging the uptake of drugs based on financial considerations are included in this systematic review |

| **Author** | **Ranji, S. R. et al** |
| --- | --- |
| **Title** | **Interventions to Reduce Unnecessary Antibiotic Prescribing**  ***A Systematic Review and Quantitative Analysis*** |
| **Year** | 2008 |
| **Reason for exclusion** | Unclear information about the evidence base of some individual studies within the review |

| **Author** | **Harvey E, Glenny AM, Kirk S, Summerbell CD** |
| --- | --- |
| **Title** | **Improving health professionals’ management and the organisation of care for overweight and obese people.**  ***Cochrane Database of Systematic Reviews* 2001** |
| **Year** | 2001 (update of 1999 study) |
| **Reason for exclusion** | The link between research evidence and practice is not sufficiently explicit. |

| **Author** | **Mathew Prior, Michelle Guerin and Karen Grimmer-Somers** |
| --- | --- |
| **Title** | **The effectiveness of clinical guideline implementation**  **strategies – a synthesis of systematic review findings** |
| **Year** | 2008 |
| **Reason for exclusion** | The link between research evidence and practice is not sufficiently explicit. |

| **Author** | **Van der Wees, P.J. et al** |
| --- | --- |
| **Title** | **Multifaceted strategies may increase implementation of physiotherapy clinical guidelines: a systematic review** |
| **Year** | 2008 |
| **Reason for exclusion** | Unclear information about the evidence base of some individual studies within the review |

| **Author** | **Akbari A, Mayhew A, Al-AlawiMA, Grimshaw J, Winkens R, Glidewell E, Pritchard C, Thomas R, Fraser C.** |
| --- | --- |
| **Title** | **Interventions to improve outpatient referrals from primary care to secondary care. *Cochrane Database of Systematic Reviews* 2008** |
| **Year** | 2008 (Update of Grimshaw 2005) |
| **Reason for exclusion** | The link between research evidence and practice is not sufficiently explicit. |

| **Author** | **Francke, A. L. et al** |
| --- | --- |
| **Title** | **Factors influencing the implementation of clinical guidelines for**  **health care professionals: A systematic meta-review** |
| **Year** | 2008 |
| **Reason for exclusion** | Overview of reviews rather than a systematic review. |

| **Author** | **Winston G. Satterlee, Robin G. Eggers and David A. Grimes** |
| --- | --- |
| **Title** | **Effective Medical Education: Insights From the Cochrane Library** |
| **Year** | 2008 |
| **Reason for exclusion** | The link between research evidence and practice is not sufficiently explicit. |

| **Author** | **Zwarenstein M, Goldman J, Reeves S.** |
| --- | --- |
| **Title** | **Interprofessional collaboration: effects of practice-based interventions on professional practice and healthcare outcomes.**  ***Cochrane Database of Systematic Reviews* 2009** |
| **Year** | 2009 (update of 2000 study) |
| **Reason for exclusion** | The link between research evidence and practice is not sufficiently explicit. |

| **Author** | **Marinopoulos, S. S et al** |
| --- | --- |
| **Title** | **Effectiveness of continuing medical education** |
| **Year** | 2007 |
| **Reason for exclusion** | The link between research evidence and practice is not sufficiently explicit. |

| **Author** | **Mansouri, M. Lockyer, J.** |
| --- | --- |
| **Title** | **A Meta-Analysis of Continuing Medical Education Effectiveness** |
| **Year** | 2007 |
| **Reason for exclusion** | The link between research evidence and practice is not sufficiently explicit. |

| **Author** | **Thomas, D. C. et al** |
| --- | --- |
| **Title** | **Continuing Medical Education, Continuing Professional Development, and Knowledge Translation: Improving Care of Older Patients by Practicing Physicians** |
| **Year** | 2006 |
| **Reason for exclusion** | The link between research evidence and practice is not sufficiently explicit. |

| **Author** | **Veloski, J. et al** |
| --- | --- |
| **Title** | **Systematic review of the literature on assessment, feedback and physicians’ clinical performance** |
| **Year** | 2006 (BEME) |
| **Reason for exclusion** | Unclear information about the evidence base of some individual studies within the review |

| **Author** | **Nagykaldi, Z. et al** |
| --- | --- |
| **Title** | **Practice Facilitators: a review of the literature** |
| **Year** | 2005 |
| **Reason for exclusion** | The link between research evidence and practice is not sufficiently explicit. |

| **Author** | **Bloom, B. S. et al** |
| --- | --- |
| **Title** | **Effects of continuing medical education on improving physician clinical care and patient health: A review of systematic reviews** |
| **Year** | 2005 |
| **Reason for exclusion** | Over-view of reviews rather than a systematic review. |

| **Author** | **Tinmouth, A. et al** |
| --- | --- |
| **Title** | **Reducing the Amount of Blood Transfused**  ***A Systematic Review of Behavioural Interventions* *to Change Physicians’ Transfusion Practices*** |
| **Year** | 2005 |
| **Reason for exclusion** | Unclear information about the evidence base of some individual studies within the review |

| **Author** | **Garg, A. X. et al** |
| --- | --- |
| **Title** | **Effects of Computerized Clinical Decision Support Systems (CDSS) on Practitioner Performance and Patient Outcomes** A Systematic Review |
| **Year** | 2005 |
| **Reason for exclusion** | The link between research evidence and practice is not sufficiently explicit. |

| **Author** | **Forsetlund L, Bjørndal A, Rashidian A, Jamtvedt G, O’Brien MA,Wolf F, Davis D, Odgaard-Jensen J, Oxman AD.** |
| --- | --- |
| **Title** | **Continuing education meetings and workshops: effects on professional practice and health care outcomes. *Cochrane Database of Systematic Reviews* 2009** |
| **Year** | 2009 (update of previous reviews 1999, 2001) |
| **Reason for exclusion** | The link between research evidence and practice is not sufficiently explicit. |

| **Author** | **Dexheimer, J.W. et al** |
| --- | --- |
| **Title** | **Prompting Clinicians about Preventive Care Measures: A Systematic Review of Randomized Controlled Trials** |
| **Year** | 2008 (update of previous 2000 study by Balas et al) |
| **Reason for exclusion** | The link between research evidence and practice is not sufficiently explicit. |

| **Author** | **Bahtsevani, C. et al** |
| --- | --- |
| **Title** | **Outcomes of evidence-based clinical practice guidelines: A systematic review** |
| **Year** | 2004 |
| **Reason for exclusion** | The link between research evidence and practice is not sufficiently explicit. |

| **Author** | **Sohn, W. et al** |
| --- | --- |
| **Title** | **Efficacy of Educational Interventions Targeting Primary Care Providers’ Practice Behaviors: an Overview of Published Systematic Reviews** |
| **Year** | 2004 |
| **Reason for exclusion** | Over-view of reviews rather than a systematic review. |

| **Author** | **Robertson, M. K. et al** |
| --- | --- |
| **Title** | **Impact studies in continuing education for health professions: update** |
| **Year** | 2003 |
| **Reason for exclusion** | The link between research evidence and practice is not sufficiently explicit. |

| **Author** | **Saillour-Glenisson, F. Michel, P.** |
| --- | --- |
| **Title** | **Individual and collective facilitators and barriers to the use of clinical practice guidelines by physicians: a comprehensive literature review** |
| **Year** | 2002 |
| **Reason for exclusion** | The link between research evidence and practice is not sufficiently explicit. |

| **Author** | **Cauffman, J. G. et al** |
| --- | --- |
| **Title** | **Randomized Controlled Trials of Continuing Medical Education: What Makes Them Most Effective?** |
| **Year** | 2002 |
| **Reason for exclusion** | The link between research evidence and practice is not sufficiently explicit. |

| **Author** | **Figueiras, A. et al** |
| --- | --- |
| **Title** | **Effectiveness of educational interventions on the improvement of drug prescription in primary care: a critical literature review** |
| **Year** | 2001 |
| **Reason for exclusion** | A significant number of studies encouraging the uptake of drugs based on financial considerations are included in this systematic review |

| **Author** | **Farmer AP, Légaré F, Turcot L, Grimshaw J, Harvey E, McGowan JL, Wolf F.** |
| --- | --- |
| **Title** | **Printed educational materials: effects on professional practice and health care outcomes. *Cochrane Database of Systematic Reviews* 2008** |
| **Year** | 2008 (update of 1997 review) |
| **Reason for exclusion** | Unclear information about the evidence base of some individual studies within the review |

| **Author** | **W. Clayton Bordley et al** |
| --- | --- |
| **Title** | **The Effect of Audit and Feedback on Immunization Delivery - A Systematic Review** |
| **Year** | 2000 |
| **Reason for exclusion** | The link between research evidence and practice is not sufficiently explicit. |

| **Author** | **Davis. D, et al** |
| --- | --- |
| **Title** | **Impact of Formal Continuing Medical Education - Do Conferences, Workshops, Rounds, and Other Traditional Continuing Education Activities Change Physician Behavior or Health Care Outcomes?** |
| **Year** | 1999 |
| **Reason for exclusion** | The link between research evidence and practice is not sufficiently explicit. |

| **Author** | **Soloman. D. H. et al** |
| --- | --- |
| **Title** | **Techniques to Improve Physicians’ Use of Diagnostic Tests. A New Conceptual Framework** |
| **Year** | 1998 |
| **Reason for exclusion** | The link between research evidence and practice is not sufficiently explicit. |

| **Author** | **Cabana, M. D. et al** |
| --- | --- |
| **Title** | **Why Don’t Physicians Follow Clinical Practice Guidelines?** A Framework for Improvement |
| **Year** | 1999 |
| **Reason for exclusion** | The link between research evidence and practice is not sufficiently explicit. |

| **Author** | **Knaup, C. et al** |
| --- | --- |
| **Title** | **Effect of feedback of treatment outcome in specialist mental healthcare: meta-analysis** |
| **Year** | 2009 |
| **Reason for exclusion** | The link between research evidence and practice is not sufficiently explicit. |

| **Author** | **Shojania KG, Jennings A,Mayhew A, Ramsay CR, EcclesMP, Grimshaw J.** |
| --- | --- |
| **Title** | **The effects of on-screen, point of care computer**  **reminders on processes and outcomes of care.. *Cochrane Database of Systematic Reviews* 2009** |
| **Year** | 2009 |
| **Reason for exclusion** | The link between research evidence and practice is not sufficiently explicit. |

| **Author** | **Guldberg, L. G. et al** |
| --- | --- |
| **Title** | **The effect of feedback to general practitioners on quality of care for**  **people with type 2 diabetes. A systematic review of the literature** |
| **Year** | 2009 |
| **Reason for exclusion** | The link between research evidence and practice is not sufficiently explicit. |

| **Author** | **Mazmanian, P. E. et al** |
| --- | --- |
| **Title** | **Continuing medical education effect on clinical outcomes. Effectiveness of Continuing Medical Education: American College of Chest Physicians Evidence-Based Educational Guidelines** |
| **Year** | 2009 |
| **Reason for exclusion** | The link between research evidence and practice is not sufficiently explicit. |

| **Author** | **Hysong, S. J** |
| --- | --- |
| **Title** | **Meta-analysis: audit and feedback features impact effectiveness on care quality** |
| **Year** | 2009 |
| **Reason for exclusion** | The link between research evidence and practice is not sufficiently explicit. |

| **Author** | **Schedlbauer, A. et al** |
| --- | --- |
| **Title** | **What Evidence Supports the Use of Computerized Alerts and Prompts to Improve Clinicians’ Prescribing Behavior?** |
| **Year** | 2009 |
| **Reason for exclusion** | The link between research evidence and practice is not sufficiently explicit. |

| **Author** | **Pearson, S. A. et al** |
| --- | --- |
| **Title** | **Do computerised clinical decision support systems for prescribing change practice? A systematic review of the literature (1990-2007)** |
| **Year** | 2009 |
| **Reason for exclusion** | The link between research evidence and practice is not sufficiently explicit. |

| **Author** | **Kawamoto, K. et al** |
| --- | --- |
| **Title** | **Improving clinical practice using clinical decision support systems: a systematic review of trials to identify features critical to success** |
| **Year** | 2005 |
| **Reason for exclusion** | The link between research evidence and practice is not sufficiently explicit. |
